# PANoptosis-based molecular clustering and prognostic signature predicts patient survival and immune landscape in colon cancer

**DOI:** 10.3389/fgene.2022.955355

**Published:** 2022-09-14

**Authors:** Xu Wang, Rui Sun, Shixin Chan, Lei Meng, Yuanmin Xu, Xiaomin Zuo, Zhenglin Wang, Xianyu Hu, Qijun Han, Longfei Dai, Tao Bai, Zhen Yu, Ming Wang, Wenqi Yang, Huabing Zhang, Wei Chen

**Affiliations:** ^1^ Department of General Surgery, The First Affiliated Hospital of Anhui Medical University, Hefei, Anhui, China; ^2^ Affiliated Chuzhou Hospital of Anhui Medical University, First People’s Hospital of Chuzhou, Chuzhou, Anhui, China; ^3^ Department of Biochemistry and Molecular Biology, Metabolic Disease Research Center, School of Basic Medicine, Anhui Medical University, Hefei, Anhui, China

**Keywords:** PANoptosis, colon cancer, prognosis, tumor microenvironment, immunotherapy

## Abstract

PANoptosis is a newly-discovered cell death pathway that involves crosstalk and co-ordination between pyroptosis, apoptosis, and necroptosis processes. However, the roles of PANoptosis-related genes (PRGs) in prognosis and immune landscape of colon cancer remain widely unknown. Here, we performed a bioinformatics analysis of expression data of nineteen PRGs identified from previous studies and clinical data of colon cancer patients obtained from TCGA and GEO databases. Colon cancer cases were divided into two PRG clusters, and prognosis-related differentially expressed genes (PRDEGs) were identified. The patient data were then separated into two corresponding distinct gene clusters, and the relationship between the risk score, patient prognosis, and immune landscape was analyzed. The identified PRGs and gene clusters correlated with patient survival and immune system and cancer-related biological processes and pathways. A prognosis signature based on seven genes was identified, and patients were divided into high-risk and low-risk groups based on the calculated risk score. A nomogram model for prediction of patient survival was also developed based on the risk score and other clinical features. Accordingly, the high-risk group showed worse prognosis, and the risk score was related to immune cell abundance, cancer stem cell (CSC) index, checkpoint expression, and response to immunotherapy and chemotherapeutic drugs. Results of quantitative real-time polymerase chain reaction (qRT-PCR) showed that LGR5 and VSIG4 were differentially expressed between normal and colon cancer samples. In conclusion, we demonstrated the potential of PANoptosis-based molecular clustering and prognostic signatures for prediction of patient survival and tumor microenvironment (TME) in colon cancer. Our findings may improve our understanding of the role of PANoptosis in colon cancer, and enable the development of more effective treatment strategies.

## Introduction

It is estimated that there are more than 1.9 million new cases of colorectal cancer (CRC). CRC also caused 935,000 deaths in 2020, accounting for approximately one-tenth of all cancer cases and deaths. Among all types of cancer, CRC ranks third in incidence rate but second in mortality (Sung et al., 2021). Patients with early colon cancer can be surgically treated. However, most patients with advanced colon cancer experience cancer recurrence and metastasis, and their 5-years survival rates are less than 10% (Bhandari et al., 2017; Doonan et al., 2017; Russo et al., 2019). With the development of chemotherapy and targeted drugs, the overall survival rate of patients with colon cancer has been significantly higher than that in the past. In recent years, progress in tumor immunotherapy and the application of immune checkpoint inhibitors have led to improvements in cancer treatment. Programmed cell death protein 1 (*PD-1*), first discovered in 1992, is a 288 amino acid protein expressed on the surface of T-cells and is involved in apoptosis (Kouo et al., 2015). In 2014, the FDA approved small cell blocking antibody (volumab) for the treatment of advanced lung cancer; volumab was further approved for the treatment of melanoma in 2015.

Cell death is a basic physiological process occurring in all organisms. Its role spans from embryonic development, organ maintenance, and aging, to coordinating immune responses and autoimmunity (Bertheloot et al., 2021). Among the proposed forms of programmed cell death (PCD), pyroptosis, apoptosis, and necroptosis are the most clearly defined. These processes involve complex molecular mechanisms responsible for the initiation, transduction, and execution of cell death (Galluzzi et al., 2018; Kesavardhana et al., 2020). Early studies on cell death have mainly focused on the unique procedures and biochemical functions under these separate mechanisms. However, recent studies have emphasized on the redundancies and crosstalk among them. PANoptosis is a newly discovered concept that highlights the crosstalk and coordination between pyroptosis, apoptosis, and necroptosis (Malireddi et al., 2019; Samir et al., 2020). Dysregulated cell death and inflammatory responses are related to tumorigenesis. Hanahan (2022) suggested that resistance to cell death is one of the basic hallmarks of cancer. Caspase-8 (*CASP8*) is a molecular switch that controls pyroptosis, apoptosis, and necroptosis (Fritsch et al., 2019). Jiang et al. reported that *CASP8* is a key protein in the crosstalk signaling pathway of PANoptosis in cancer (Jiang et al., 2021). A recent study (Karki et al., 2021a) showed that *ZBP1* activates PANoptosis through *RIPK3* signaling, *ADAR1* negatively regulates *ZBP1*-mediated apoptosis, and blocking ADAR1 activity contributes to apoptosis and inhibits tumorigenesis. Acquiring more knowledge about the effects of PANoptosis on cancer is vital for developing new strategies for cancer therapy.

Many studies have focused on constructing tumor classifications and prognostic signatures based on gene and non-coding RNA expression levels to predict the survival and immune landscape of patients with cancer. In recent years, genes and non-coding RNA related to various forms of cell death have been explored in many studies, including autophagy-, ferroptosis-, pyroptosis-, necroptosis-related genes and lncRNAs. Wang et al. (2021) identified six autophagy-related genes and developed a prognostic signature that can independently predict prognosis and reflect overall immune response intensity in the colon cancer microenvironment. Nie et al. (2021) constructed a novel ferroptosis-related gene signature to predict the prognosis and immune status of patients with colon cancer. Song et al. (2021) used pyroptosis-related genes to identify molecular subtypes and develop a prognostic model for characterizing tumor microenvironment infiltration in colorectal cancer. A recent study (Zhao et al., 2021) also used necroptosis-related lncRNAs to predict prognosis and identify molecular classifications to distinguish between cold and hot tumors in gastric cancer. However, genes related to the crosstalk and coordination between different types of cell death have not been well studied.

Our study demonstrated that PANoptosis-based molecular clustering and prognostic signatures could predict prognosis and the intratumoral immune landscape in patients with colon cancer. First, 951 colon cancer patients were divided into two discrete PRG clusters based on expression levels of PANoptosis-related genes (PRGs). Patients were then classified into two clusters based on differentially expressed genes (DEGs) identified from the two PRG clusters. A risk score was further calculated, and a prognostic signature was established to predict overall survival (OS) and response to immunotherapy in colon cancer patients.

## Materials and methods

### Data acquisition

Gene expression data (fragments per kilobase million, FPKM) and relevant clinical information of colon cancer patients were retrieved from the TCGA (https://portal.gdc.cancer.gov) and GEO (https://www.ncbi.nlm.nih.gov/geo/, ID: GSE39583) databases. Nineteen PANoptosis-related genes (PRGs) were identified from prior studies (Malireddi et al., 2019; Karki et al., 2020; Malireddi et al., 2020; Samir et al., 2020; Briard et al., 2021; Jiang et al., 2021; Lee et al., 2021; Place et al., 2021; Nguyen and Kanneganti, 2022), and the genes are listed in Supplementary Table S1. The FPKM values of TCGA colon adenocarcinoma (COAD) were transformed into transcripts per kilobase million (TPM) using the R software (version 4.1.1). TCGA and GEO data were combined and batch effects were eliminated using the Combat algorithm of the *sva* R package. Patients with no follow-up data or incomplete clinical information were excluded from the study. Finally, 951 patients were included in the study. The clinical information of patients with colon cancer is presented in Supplementary Table S2.

### Consensus clustering analysis of PANoptosis-related genes

A consensus clustering analysis based on PRGs expression was performed to investigate the connections between PRGs and colon cancer subtypes using the *ConsensusClusterPlus* R package. The classification with the highest intragroup correlations and the lowest intergroup correlations was identified. The prognosis of the two clusters was compared using the Kaplan-Meier (KM) method and log-rank test. Principal component analysis (PCA) was performed using the stats R package. Differences in clinical features between two clusters were analyzed using the Wilcoxon test, and DEGs between two clusters were identified with the criteria of |log fold change (FC)| >1 and *p*-value < 0.05, using *limma* package. To explore the differences in biological processes between the two PRG clusters, we performed gene set variation analysis (GSVA) using gsva R package. Single-sample gene set enrichment analysis (ssGSEA) was used to calculate the scores of infiltrating immune cells and evaluate the activity of immune-related functions.

### Gene oncology and kyoto encyclopedia of genes and genomes analyses

To understand the biological functions and pathways related to the DEGs, Gene Ontology (GO) and Kyoto Encyclopedia of Genes and Genomes (KEGG) analyses were performed using the *ggplot2*, *Bioconductor*, and *org. Hs.eg.db* R packages. *p*-values and *q*-values < 0.05 were considered statistically significant.

### Construction of the PANoptosis-related prognostic signature

Prognosis-related DEGs (PRDEGs) were selected using a univariate Cox regression analysis. To identify additional PANoptosis-related genes for signature construction. We classified patients into two distinct clusters based on the expression of PRDEGs. Survival time, clinical features, and PRG expression were compared between the two gene clusters, and DEGs between gene clusters A and B were identified. Finally, seven genes were included to construct the prognostic signature after least absolute shrinkage and selection operator (LASSO) regression analysis and multivariate Cox regression analysis using the *survival*, *survminer*, and *glmnet* R packages. The risk score was calculated based on the expression levels of the seven genes, and the patients were divided into high- and low-risk groups using the median risk score. The KM analysis was used to evaluate survival differences between high-risk and low-risk groups, and the receiver operating characteristic (ROC) and area under the curve (AUC) were used to test the prediction efficiency of the risk score. A nomogram model was developed based on risk scores and clinical factors. Calibration graphs were constructed to show the differences between the nomogram-predicted and actual survival rates of colon cancer patients.

### Evaluation of the tumor microenvironment between the high- and low-risk groups

To better understand the relationship between the risk score and tumor microenvironment (TME), CIBERSORT was used to quantify the abundance of infiltrating immune cells in heterogeneous samples from the high- and low-risk groups. The correlation between the abundance of immune cells and the seven genes was analyzed. TME scores, including stromal, immune, and ESTIMATE scores, of high- and low-risk groups were also compared using the Wilcoxon signed-rank test.

### Analyses of mutations, microsatellite instability and cancer stem cell index between high- and low-risk groups

To explore the gene mutations in colon cancer patients in high- and low-risk groups, we generated the mutation annotation format (MAF) using the *maftool*s R package. The tumor mutation burden (TMB) score of patients was calculated, and the correlation between the risk score and TMB was analyzed using the Spearman method. Furthermore, we analyzed the association between risk groups and MSI and CSC index.

### Response to immunotherapy and chemotherapeutic drugs

The Cancer Immunome Atlas (TCIA, https://tcia.at/) is a dataset containing TCGA data of 20 solid cancers and more than 8,000 tumor samples. It can be used to calculate the immunophenotypic score (IPS) of tumor samples to predict the response to cytotoxic T lymphocyte antigen-4 (CTLA-4) and programmed cell death protein 1 (PD-1) blockers (Charoentong et al., 2017). IC50 is half of the maximum inhibitory concentration and represents the concentration of the drug required for 50% inhibition of cancer cells. To determine the relationship between the risk score and response to immunotherapy and chemotherapeutic drugs, checkpoint expression, immune subtypes, IPS of tumor samples, and the IC50 of drugs in the two risk groups were calculated and compared.

### The verification of LGR5, VSIG4, GZMB, and ITLN1 by quantitative real-time polymerase chain reaction

Ten pairs of colon cancer and corresponding non-tumor tissues were collected from colon cancer patients in The First Affiliated Hospital of Anhui Medical University, the samples were preserved at −80°C. The study was approved by the Ethics Committee of The First Affiliated Hospital of Anhui Medical University. All participants signed an informed consent form. Total RNA was extracted using The HiPure Universal RNA Kit (Shanghai, Magen). Extracted RNA was reverse transcribed into cDNA using the RevertAid First Strand cDNA Synthesis Kit (Thermo Fisher Scientific, United States). The concentration of cDNA was measured using TB Green Premix Ex Taq II (GenStar, China) under the LightCycler480 System (Applied Biosystems, Waltham, MA, United States). The relative expression levels were computed using the 2^−ΔΔCt^ method, normalizing with *36B4*. The primer sequences for PCR amplification are shown in Supplementary Table S3. The differences of expression levels between colon cancer tissues and adjacent non-cancer tissues were compared using unpaired *t*-tests. The graphs were drawn using GraphPad Prism software (version 9.0.0).

## Results

### Landscape of genetic variation of PANoptosis-related genes in colon cancer

**FIGURE 1:**
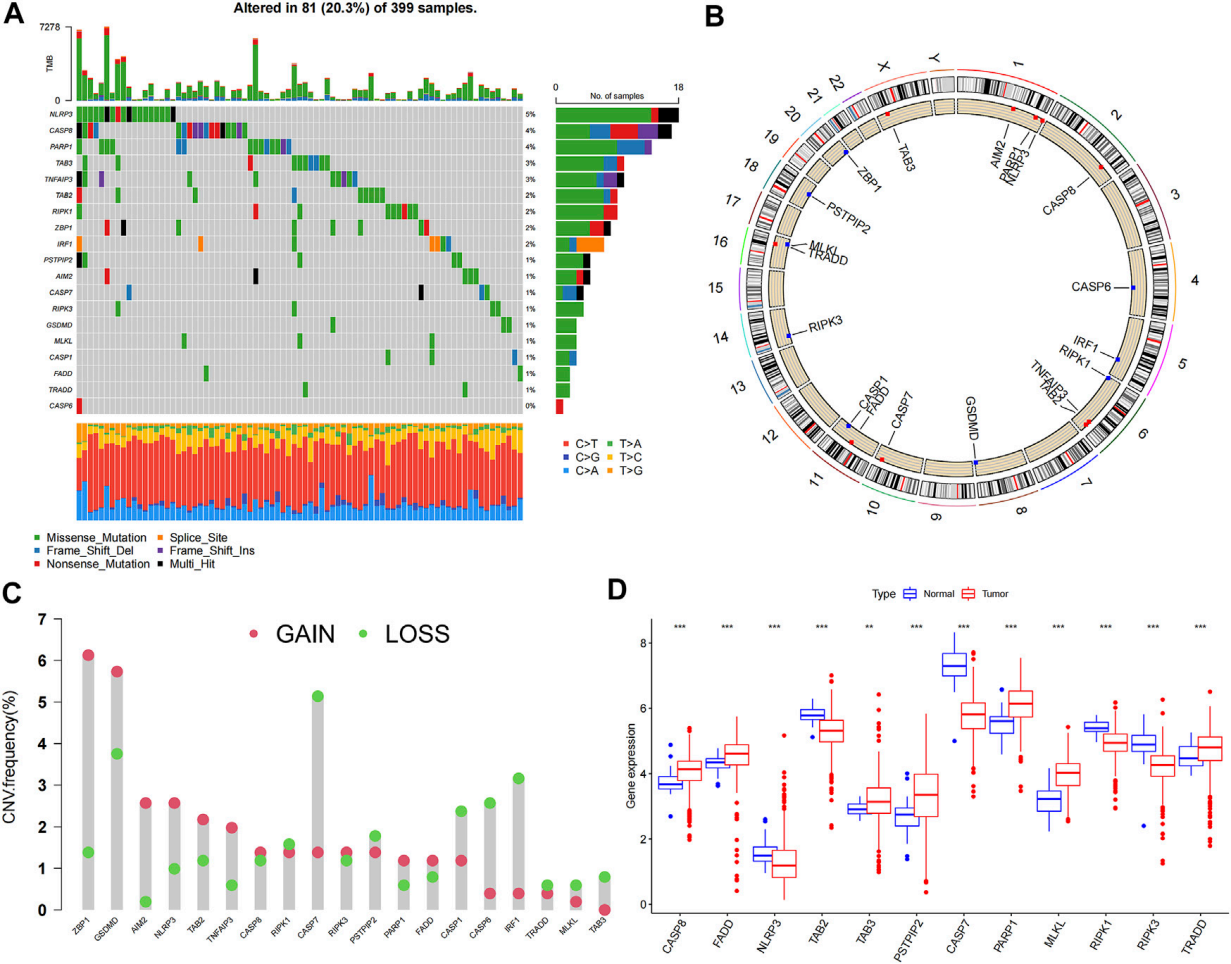
Genetic and transcriptional alterations of 19 PRGs in colon cancer. **(A)** Mutation frequencies of 19 PRGs in colon cancer patients from TCGA cohort; **(B)** Locations of CNV alterations in PRGs on 23 chromosomes; **(C)** Frequencies of CNV gain, loss, and non-CNV among PRGs; **(D)** Expression levels of PRGs between normal and tumor samples. **p < 0.01; ***p < 0.001.

Expression data of 457 COAD patients were downloaded from the TCGA database, and expression levels of PRGs were compared between 41 normal and 473 tumor samples. Nineteen PRGs from previous studies were included in this study. The somatic mutation incidence in the 19 PRGs of colon cancer patients is shown in Figure 1A; 81 (20.3%) of the 399 samples had altered PRGs. Among the 19 PRGs, *NLRP3* showed the highest mutation frequency. Figure 1B shows the locations of the CNV alterations in PRGs on their chromosomes. Twelve PRGs were differentially expressed in colon cancer samples compared with their expression in normal samples. Somatic copy number alterations of the 19 PRGs were analyzed; *ZBP1, GSDMD, AIM2*, and *NLRP3* had the highest copy number variation (CNV), whereas *CASP7, CASP1, CASP6*, and *IRF3* showed significant CNV decreases (Figure 1C). Among these 12 PRGs, seven genes were upregulated in tumor samples, including *CASP8, FADD, TAB3, PSTPIP2, PARP1, MLKL,* and *TRADD*, whereas the other five genes, including *NLRP3, TAB2, CASP7, RIPK1,* and *RIPK3,* were downregulated in tumor samples (*p* < 0.05) (Figure 1D).

### Identification of PANoptosis-related gene clusters in colon cancer

**FIGURE 2:**
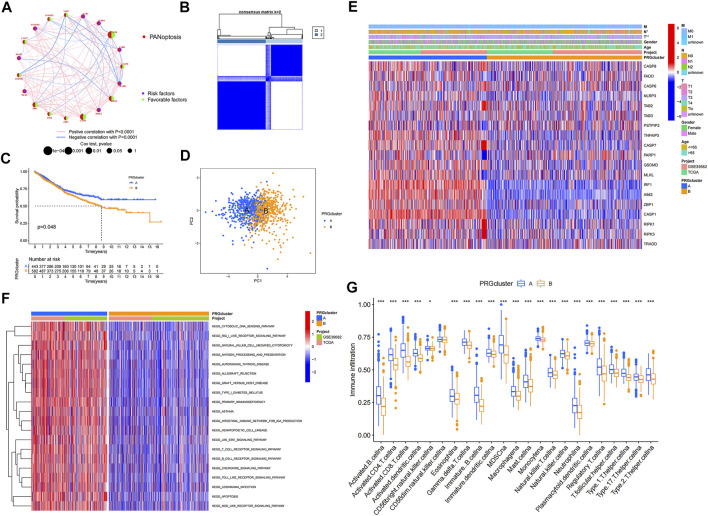
PRGclusters and clinical characteristics between colon cancer samples in two clusters. Relationship of tumor microenvironment in two PRGclusters. **(A)** Interactions among PRGs in colon cancer. The lines among the PRGs represents their interactions. Blue and red represent negative and positive correlations. **(B)** Two PRGclusters were defined using consensus clustering analyses. **(C)** KM curve indicated that PRGcluster a had longer survival time than PRGcluster B (*p* = 0.048). **(D)** PCA showed good distiction between two PRGclusters. **(E)** Heatmaps showed the relationship between PRGclusters and clinical features and PRGs expression in colon cancer patients. **(F)** GSVA showed the enriched pathways in PRGclusters. **(G)** ssGSEA investigated the differences of immune cell infiltration between two clusters. *p < 0.05; **p < 0.01.

To explore the interactions between the 19 PRGs and their prognostic significance, a network was constructed, as shown in Figure 2A. Kaplan–Meier curves of the relationship between PRGs expression and the prognosis of colon cancer patients were shown in Supplementary Figure S1. Consensus clustering analysis was performed to explore the relationship between PRG expression and tumor classification (Supplementary Figure S2). Clusters with the highest intragroup correlations and lowest intergroup correlations were identified. By increasing the clustering variable (*k*), we found that when *k* = 2, classification met the standard. Colon cancer patients were divided into two PRG clusters (A and B) based on PRG expression levels (Figure 2B). As shown in Figure 2C, patients in PRG cluster A had a significantly longer survival time than those in cluster B (*p* = 0.048). PCA showed a satisfactory separation between PRG cluster A and B (Figure 2D). Figure 2E shows the association between PRG clusters and clinical features and PRG expression in colon cancer patients. Tumor infiltration and lymph node metastasis correlated with PRG clusters (*p* < 0.05). GSVA showed that PRG cluster A was significantly enriched in immune-related pathways, including natural killer cell-mediated cytotoxicity, antigen processing and presentation, primary immunodeficiency, B cell, and T-cell receptor signaling pathways (Figure 2F). To evaluate the differences in immune cell infiltration between the two clusters, ssGSEA was performed, and the results showed that PRG cluster A had higher immune cell infiltration levels, including those of activated B cells, activated CD4 + T-cells, activated CD8 + T-cells, activated dendritic cells, macrophages, mast cells, and natural killer cells (Figure 2G).

### Identification of gene clusters based on differentially expressed genes

**FIGURE 3:**
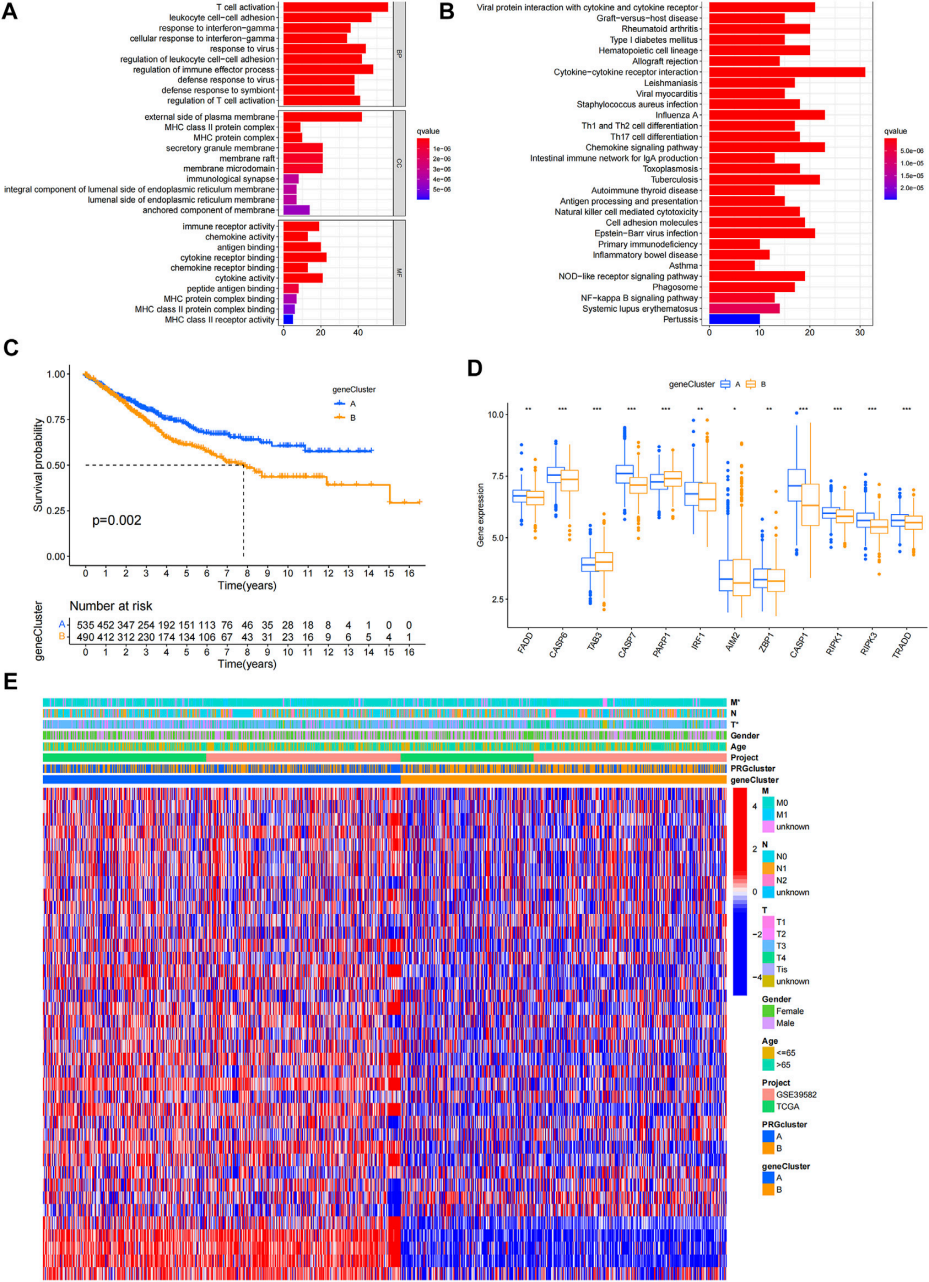
Identification of geneclusters based on DEGs. **(A–B)** GO and KEGG analyses showed the relevant biological processes (BP), cellular components (CC), molecular functions (MF) and pathways. **(C)** Heatmap showed the association between genecluster and clinical features. **(D)** KM curves showed genecluster A had a more favorable prognosis. **(E)** Expression levels of PRGs in two geneclusters. *p < 0.05; **p < 0.01; and ***p < 0.001.

DEGs were identified, and GO and KEGG analyses showed the relevant biological processes (BP), cellular components (CC), molecular functions (MF), and pathways (Figures 3A,B). These DEGs were mainly related to the BP of T cell activation, leukocyte cell-cell adhesion, and response to interferon-gamma, and were correlated with the CC such as the external side of the plasma membrane, major histocompatibility complex (MHC) class II protein complex, and MHC protein complex. Furthermore, they were involved in the MF of immune receptor activity, chemokine activity, and antigen binding. According to KEGG analysis, these DEGs participate in certain cancer-related pathways, including the chemokine signaling pathway, NOD-like receptor signaling pathway, and NF-κB signaling pathway. PRDEGs were identified using univariate Cox regression analysis. Patients were then divided into two clusters (gene cluster A and gene cluster B) based on PRDEG expression (Supplementary Figure S3; Figure 3C) shows that cluster A had higher survival rates than cluster B (*p* = 0.002). In Figure 3D, the boxplot shows that *FADD, CASP6, CASP7, IRF1, AIM2, ZBP1, CASP1, RIPK1, RIPK3,* and *TRADD* were upregulated in cluster A, whereas *TAB3* and *PARP1* were downregulated in cluster B (*p* < 0.05). The heatmaps show the association between gene clusters and clinical features and between PRDEGs expression and PRG clusters. Furthermore, gene clusters were significantly related to tumor infiltration and metastasis (*p* < 0.05) (Figure 3E).

### Development abd validation of the PANoptosis-related prognostic signature

**FIGURE 4:**
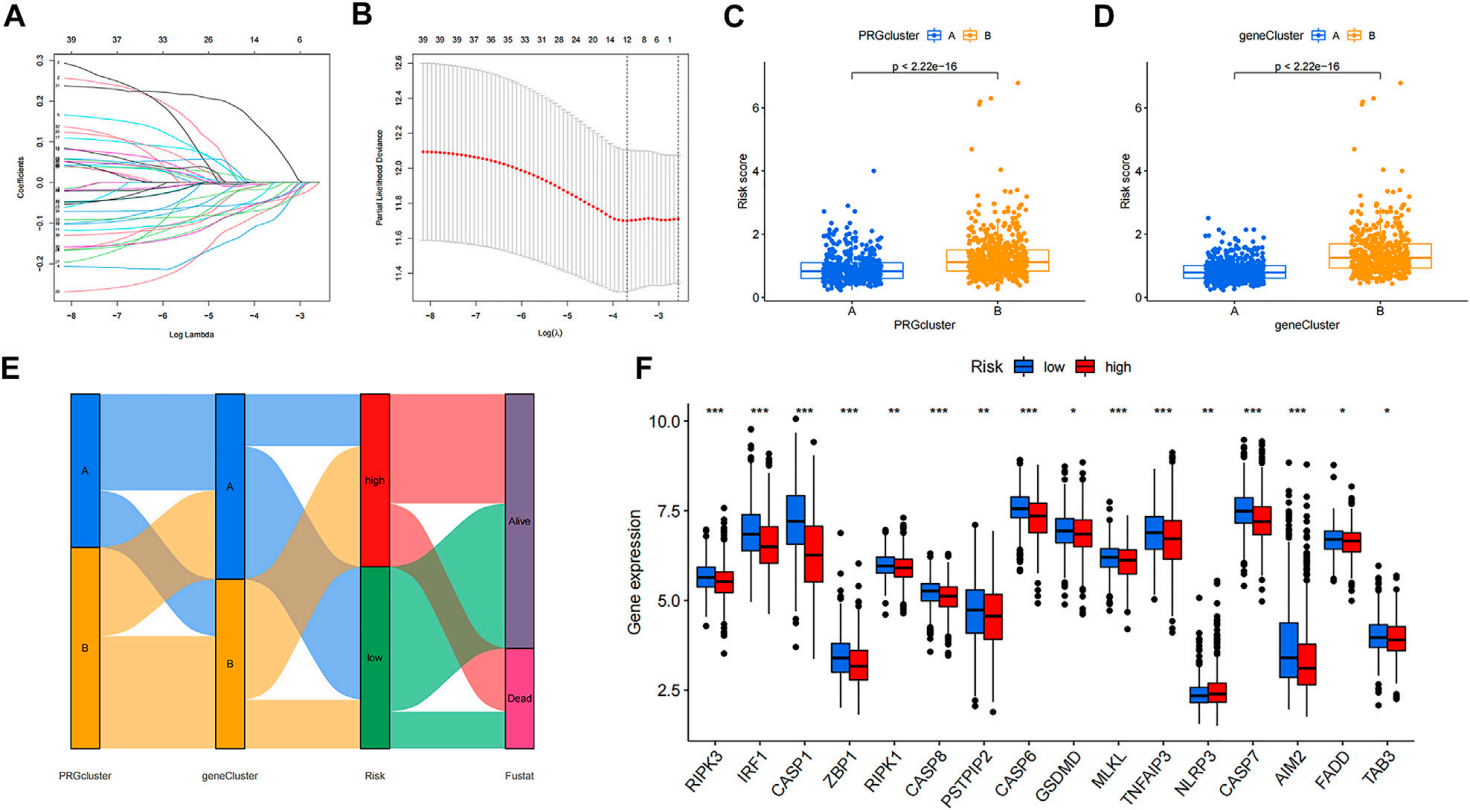
Identification of 7 genes for calculating the risk score and the relationship between molecular classifications, PRG expression levels and the risk score. **(A–B)** The LASSO regression analysis and partial likelihood deviance on the prognostic genes. **(C–D)** Association between risk score and molecular classifications. **(E)** Sankey plot showed the correlation between molecular classifications, risk groups and survival status in colon cancer patients. **(F)** Expression levels of PRGs in two risk groups. *p < 0.05; **p < 0.01; and ***p < 0.001.

**FIGURE 5:**
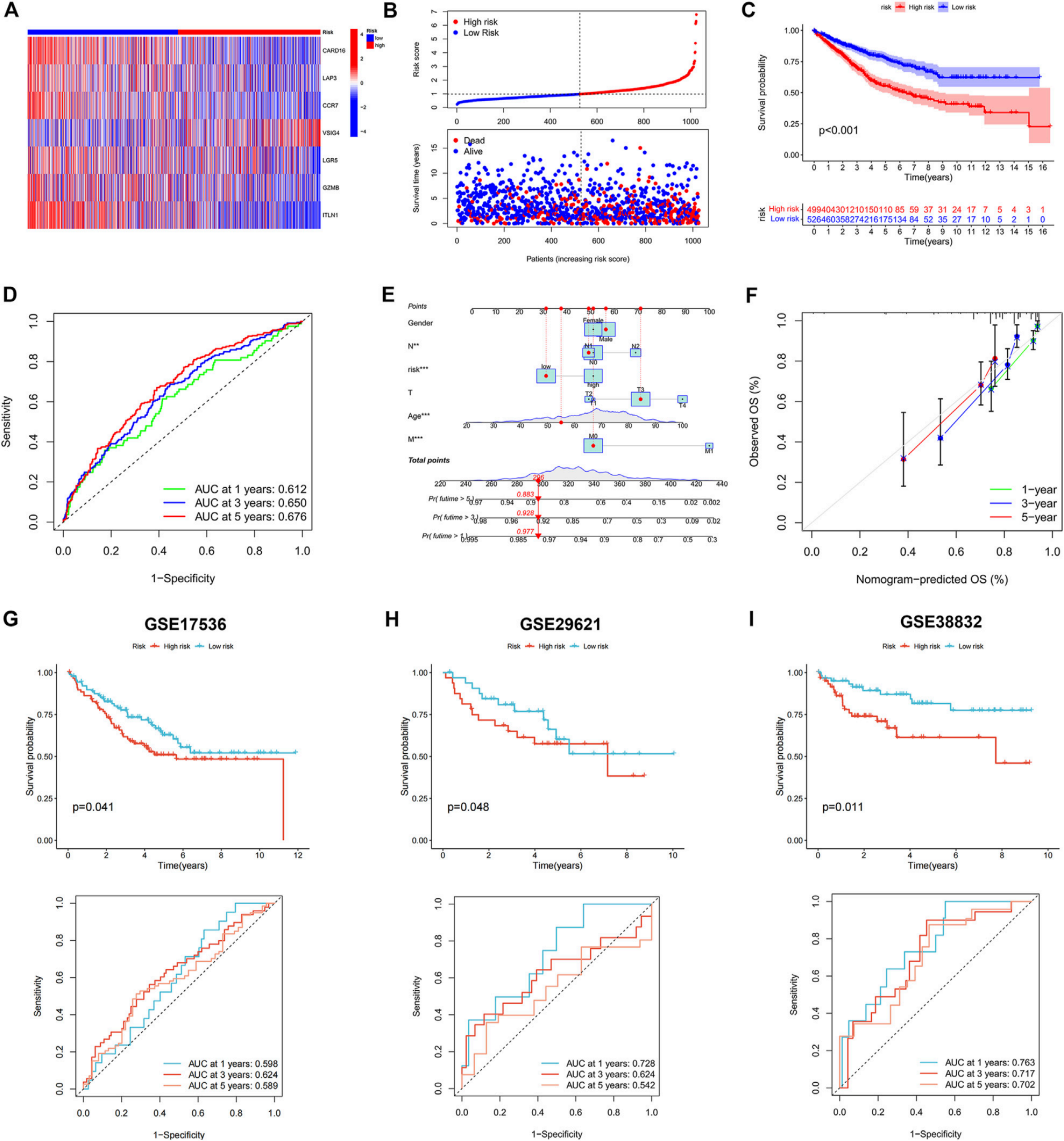
Construction and validation of the prognostic signature. **(A)** Heatmap showed the expression of 7 genes in two risk groups. **(B)** Risk score and survival outcome of each case. **(C)** KM curve showed that patients in high-risk group had a worse prognosis. **(D)** The AUC for 1-, 3- and 5-years survival were 0.612, 0.650, and 0.676, respectively. **(E)** Nomogram using risk score and other clinical features were constructed for predicting survival of colon cancer patients. **(F)** Calibration graphs investigated that the actual survival rates of colon cancer patients were close to the nomogram-predicted survival rates. The KM and ROC methods were used to evaluate the efficiency of the risk score at predicting patient survival in GSE17536 **(G)**, GSE29621 **(H)**, and GSE38832 **(I)** CRC datasets.

LASSO and Cox regression analyses were performed to screen for prognosis-related DEPRGs (Figures 4A,B). After selection, seven genes were included in the calculation of the risk score based on the following formula: Risk score = $\overset{n}{\underset{i=1}{\sum }}\beta i*\lambda i$ , where n represents the number of genes included to construct the signature, and βi and λi represent the regression coefficient and gene expression value, respectively. Boxplots showed that PRG cluster B and gene cluster B had higher risk scores than PRG cluster A and gene cluster A (Figures 4C,D). A Sankey diagram showed the associations among PRG cluster, gene cluster, risk groups, and survival status (Figure 4E). Fifteen of the nineteen PRGs were differentially expressed between the high- and low-risk groups (Figure 4F). Seven genes were used to construct the prognostic signature; Figure 5A shows the expression differences of these seven genes between the two risk groups. Based on the risk score, patients were divided into high- and low-risk groups, and patients with higher risk scores had a higher risk of mortality (Figure 5B). As shown in Figure 5C, the KM curve was plotted to show the survival differences between the two groups. Patients in the high-risk group had a significantly lower probability of survival than those in the low-risk group (*p* < 0.001). ROC curves were drawn to test the prediction efficiency of the risk score, and the AUCs for one-, three-, and 5-years survival were 0.612, 0.650, and 0.676, respectively (Figure 5D). The results of the risk score in TCGA (Supplementary Figure S4) and GSE39582 (Supplementary Figure S5) cohorts were also shown. The risk score and other clinical features were used to construct a nomogram model (Figure 5E). Calibration plots showing the differences between the nomogram-predicted and actual survival probabilities of colon cancer patients showed that the predicted survival probabilities were close to the actual survival probabilities (Figure 5F), indicating that this nomogram model accurately predicted the survival of colon cancer patients. The results for three independent validation cohorts, which were GSE17536 (Figure 5G, *p* = 0.041, 1-year AUC = 0.598, 3-years AUC = 0.624, 5-years AUC = 0.589), GSE17537 (Figure 5H, *p* = 0.048, 1-year AUC = 0.728, 3-years AUC = 0.624, 5-years AUC = 0.542), and GSE29621 (Figure 5I, *p* = 0.011, 1-year AUC = 0.763, 3-years AUC = 0.717, 5-years AUC = 0.702), revealed that the risk score could efficiently predict patient survival.

### Comparative evaluation of the tumor microenvironments of high- and low-risk groups

**FIGURE 6:**
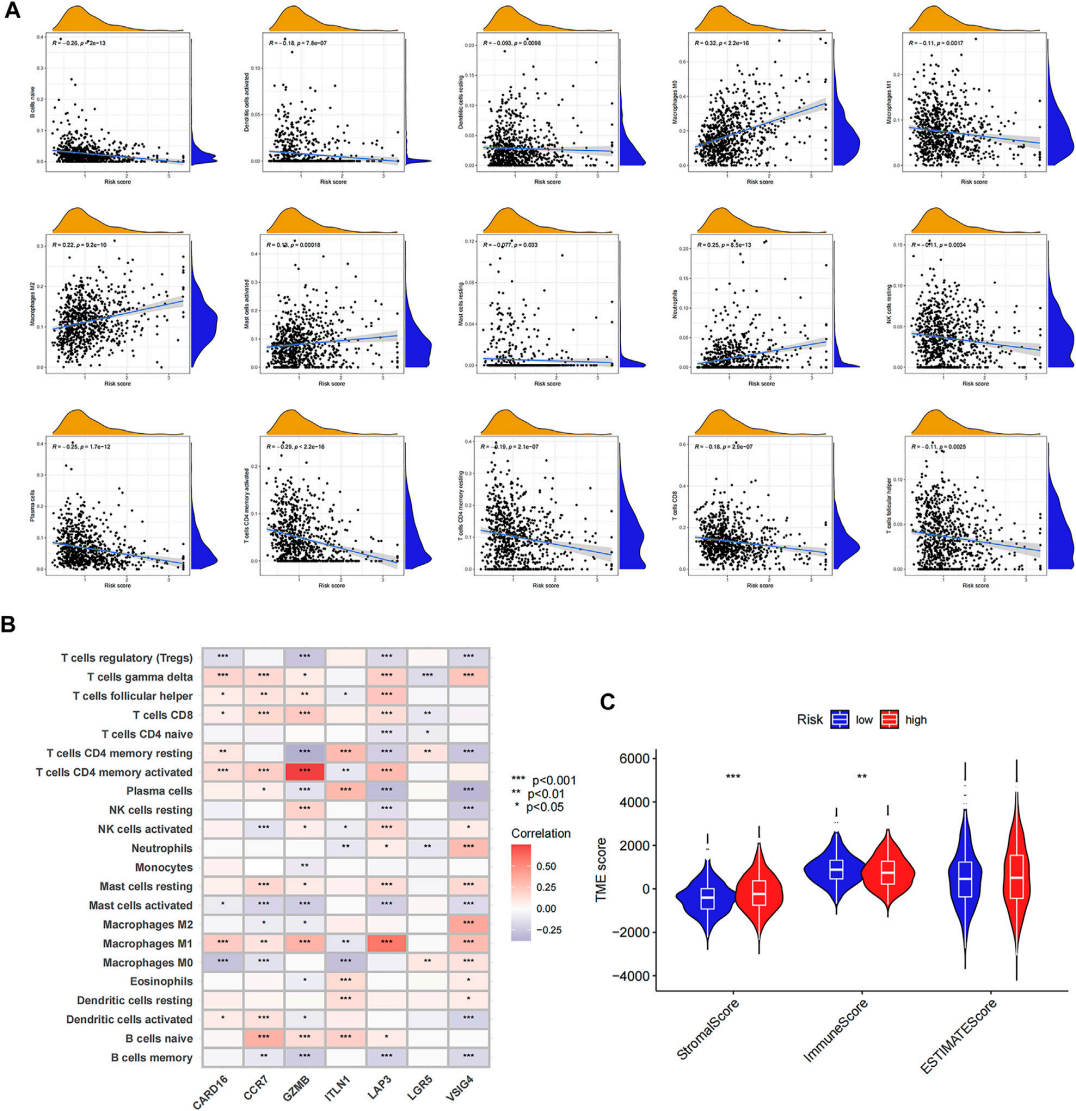
Evaluation of tumor microenvironment in high- and low-risk groups. **(A)** Relationship between risk score and different immune cell types. **(B)** Correlation between the abundance of immune cells and seven genes in the prognostic signature. **(C)** Correlation between risk score and immune-related scores. *p < 0.05; **p < 0.01; and ***p < 0.001.

Figure 6Ashows the correlation between the risk score and immune cell abundance: M0 macrophages, M2 macrophages, activated mast cells, and neutrophils were positively related to the risk score, while naive B cells, activated dendritic cells, resting dendritic cells, M1 macrophages, resting mast cells, resting NK cells, plasma cells, activated memory CD4 + T-cells, resting memory CD4 + T-cells, CD8 + T-cells, and follicular helper T-cells were negatively related to the risk score. The relationship between the abundance of immune cells and seven genes in the prognostic signature was also evaluated (Figure 6B). The TME scores in the two risk groups were calculated, and the high-risk group was found to have a higher stromal and lower immune score (Figure 6C).

### Comparative analysis of mutations, microsatellite instability and cancer stem cell index in high- and low-risk groups

**FIGURE 7:**
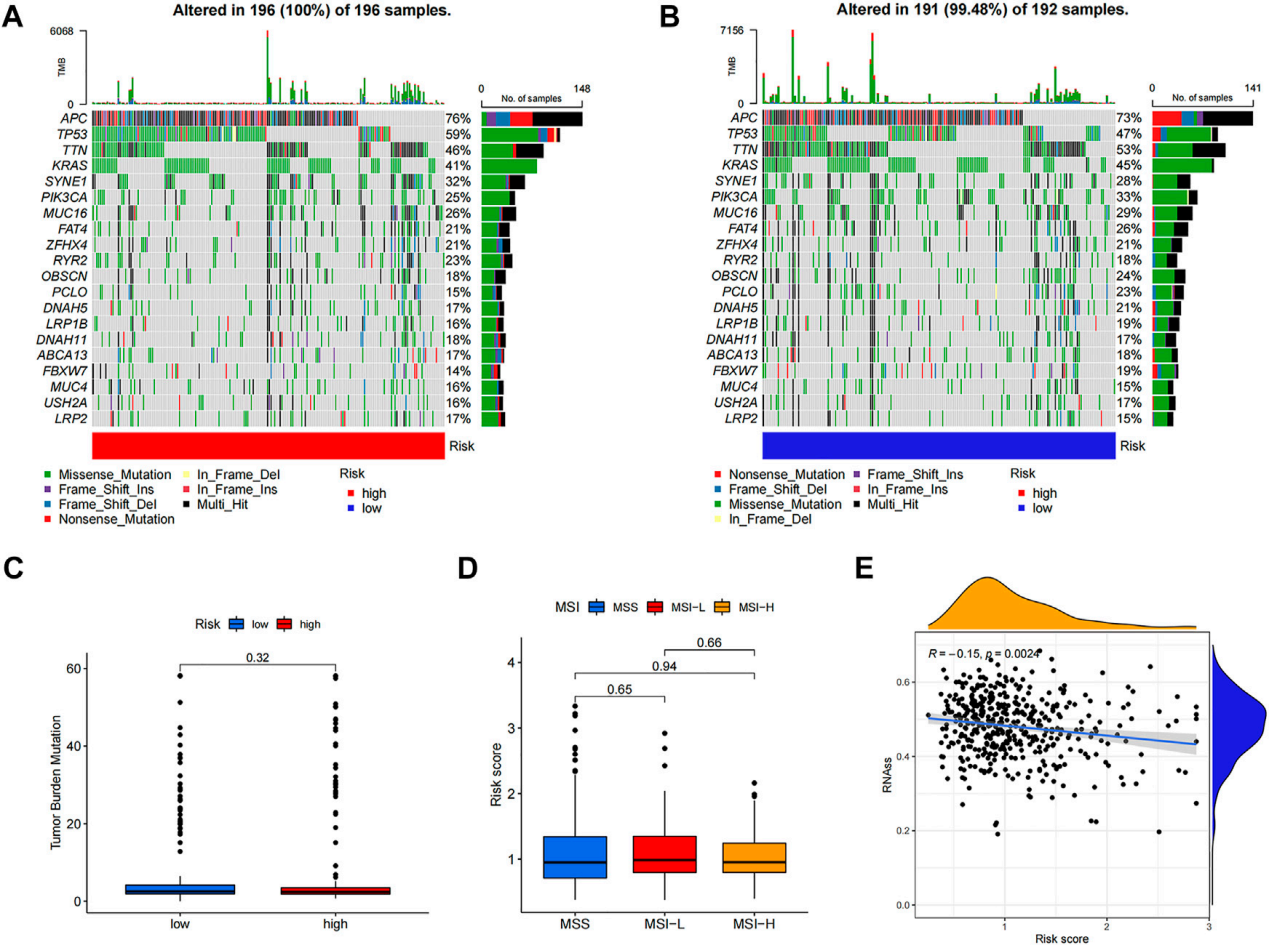
Comprehensive analyses of risk score in colon cancer. The somatic gene mutations in high-risk group **(A)** and low-risk group **(B)**. TMB **(C)** and MSI **(D)** did not show significant correlations with risk score while CSC **(E)** negatively correlated with risk score.

Differences in somatic mutations between the two risk groups of colon cancer patients were analyzed; the five most mutated genes in the high- and low-risk groups were *APC, TP53, TTN, KRAS*, and *SYNE1* (Figures 7A,B). TMB (Figure 7C) and MSI (Figure 7D) did not show a significant relationship with the risk score, while CSC (Figure 7E) was negatively correlated with the risk score (*R* = −0.15, *p* < 0.01).

### Response to immunotherapy and chemotherapeutic drugs

**FIGURE 8:**
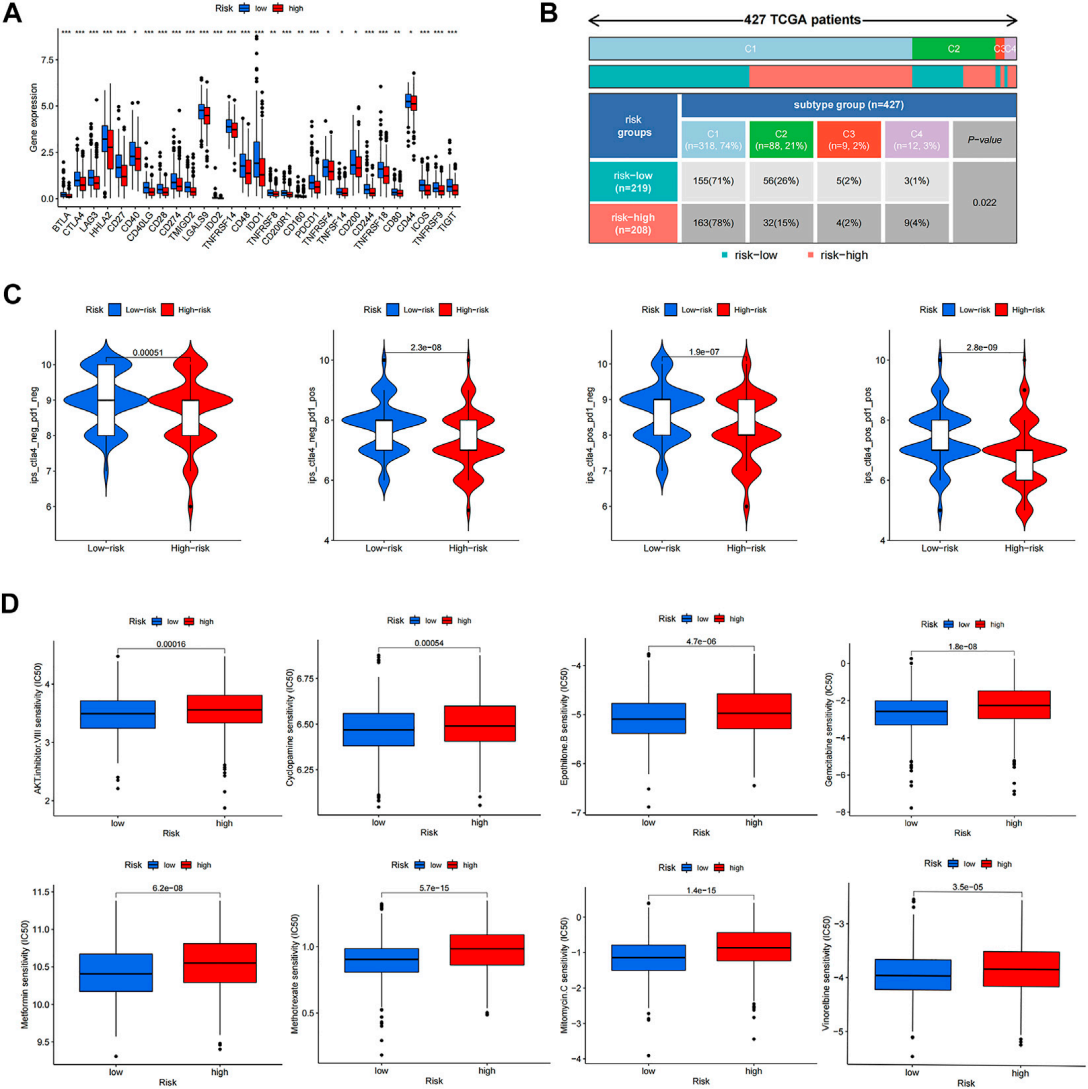
Response to anti-tumor tharapy of colon cancer patients in two risk groups. **(A)** The differences of immune checkpoint gene expression in high-risk and low-risk groups. **(B)** Heatmap and table showing the distribution of colon cancer immune subtypes between two risk groups. **(C)** Violin plots showed the relationship between IPSs and risk groups. **(D)** Eight therapeutic drugs showed significant IC50 differences. *p < 0.05; **p < 0.01; and ***p < 0.001.

To analyze the ability of risk score to predict potential checkpoint blockade therapy, boxplots were drawn to show the differences in immune checkpoint gene expression between the high- and low-risk groups (Figure 8A). Checkpoint genes, including *CTLA4, LAG3, ID O 2, CD274*, and *PDCD1*, had higher expression levels in low-risk groups. In Figure 8B, cluster1 (C1), C2, C3, and C4 represent wound healing, IFN-gamma dominant, inflammatory, and lymphocyte depleted immune subgroups, respectively (Thorsson et al., 2019). The results showed that C3 samples were almost equally distributed between the two groups, but there were more C1 and C4 samples and fewer C2 samples in the high-risk subgroup than in the low-risk subgroup. Violin plots showed the relationship between IPSs and risk groups; a higher IPS represented a better response to *PD-1* and *CTLA-4* blockers (Figure 8C). We also found that eight drugs had lower IC50 values in the high-risk group, including bexarotene, bicalutamide, dasatinib, doxetacel, elesclomol, imatinib, midostaurin, and pazopanib (Figure 8D).

### Validating expression levels of LGR5, VSIG4, GZMB, and ITLN1 *via* quantitative real-time polymerase chain reaction

**FIGURE 9:**
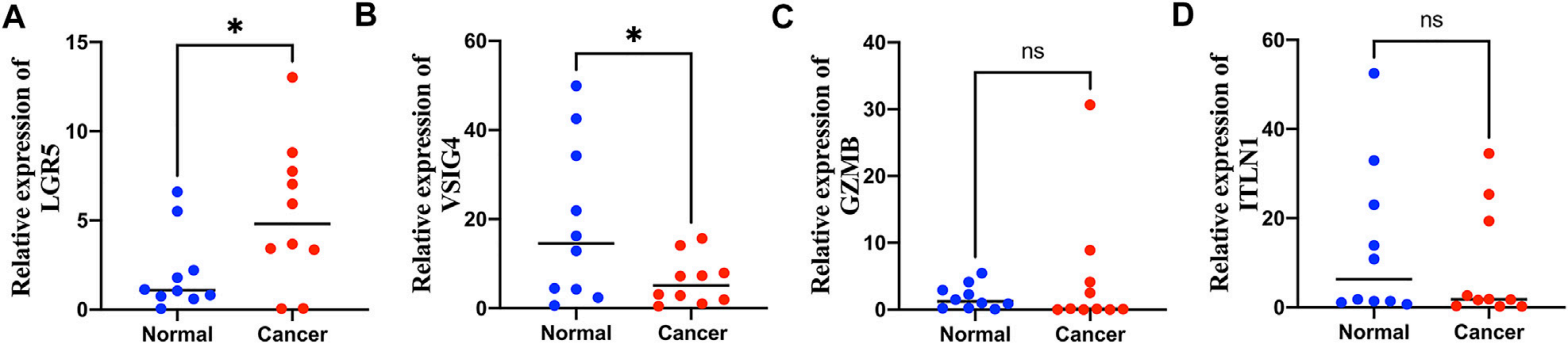
Quantitative real-time polymerase chain reaction (qRT-PCR) analyses of LGR5 **(A)**, VSIG4 **(B)**, GZMB **(C)** and ITLN1 **(D)** expression in 10 pairs of colon cancer tissues and adjacent non-cancer tissues. *p < 0.05; ns *p* > 0.05.

Among the seven genes in the prognostic signature, *LGR5*, *VSIG4*, *GZMB,* and *ITLN1* were significantly differentially expressed in colon cancer samples from GEPIA database (Supplementary Figure S6). Expression levels of *LGR5*, *VSIG4*, *GZMB,* and *ITLN1* were tested in colon cancer and adjacent normal tissues *via* qRT-PCR method. Expression of *LGR5* was significantly higher in tumor tissues (Figure 9A) while *VSIG4* had higher expression levels in normal tissues (*p* < 0.05) (Figure 9B). There was no significant differences in the expression levels of GZMB (Figure 9C) and ITLN1 (Figure 9D) between normal and tumor samples.

## Discussion

Cell death usually does not occur independently but in a mixed form because cells can undergo extensive crosstalk under pathological conditions (Zheng and Kanneganti, 2020; Karki et al., 2021b). Previous research (Karki et al., 2021b) suggests that there exists a mixed form of cell death involving pyroptosis, apoptosis, and necroptosis, called PANoptosis. In recent years, many studies have investigated the effects of different forms of cell death on various human diseases, especially malignant tumors. Some studies have revealed molecular classifications of tumors and constructed prognostic models based on genes or non-coding RNAs relevant to different forms of cell death. However, the effects of PANoptosis in colon cancer have not been well studied.

In this study, 19 PRGs were identified. In previous studies, most of these 19 PRGs were found to be related to CRC. Yin et al. (2010) suggested that overexpression of the exogenous *FADD* gene can significantly improve the apoptosis-inducing effect of 5-fluorouracil on colorectal adenocarcinoma cells. Shi et al. (2021) demonstrated that low *NLPR3* expression is related to a better prognosis of CRC. Li et al. (2020) showed that *TAB3* was upregulated in CRC tissues and promoted CRC cell growth. It was also found that *AIM2* inhibits CRC cell proliferation and migration (Xu et al., 2020). Colon cancer cases from the TCGA and GEO databases were divided into two distinct PRG clusters. PRG cluster A had a better prognosis than PRG cluster B. Tumor infiltration and lymph node metastasis were correlated with the PRG clusters. Results of GSVA and ssGSEA showed that PRG cluster A was significantly enriched in immune-related pathways and had higher immune cell infiltration levels. Tumor-infiltrating immune cells can affect the response to anti-checkpoint blockade. Furthermore, tumor-infiltrating CD4 + T-cells can upregulate programmed cell death protein 1 (*PD-1*), T-cell immunoglobulin and mucin domain-3 (*TIM-3*), cytotoxic T lymphocyte-associated protein-4 (*CTLA-4*), and lymphocyte-activation-gene-3 (*LAG-3*) (Toor et al., 2019). PRDEGs between two PRG clusters were also identified, and patients were classified into two distinct clusters. GO and KEGG analyses revealed that these PRDEGs were associated with certain cancer-related biological functions and pathways, indicating that these PRDEGs were potentially associated with malignant tumors. Gene cluster A had a longer survival time than gene cluster B, and the two clusters were correlated with tumor infiltration and metastasis.

LASSO and multivariate Cox regression analyses were used to screen genes to construct a prognostic signature. Finally, the risk score was calculated based on the expression levels of *CARD16, LAP3, CCR7, VSIG4, LGR5, GZMB*, and *ITLN1*. Some of these seven genes have been found to be associated with various types of malignant tumors. *LAG3* can promote glioma progression by regulating the proliferation, migration, and invasion of glioma cells (He et al., 2015), and inhibition of *LAG3* suppresses the invasion of ovarian cancer (Wang et al., 2015). Bill et al. (2022) suggested that *CCR7* plays distinct roles in directing tumor cells to the lymph nodes, skin, and central nervous system. Zhu et al. (2018) demonstrated that downregulated VSIG4 expression was related to poor prognosis in hepatocellular carcinoma patients with hepatitis B infection. *LGR5* has been identified as a strong cancer stem cell biomarker in CRC (Kamakura et al., 2022). These results suggest that these seven genes could serve as potential biomarkers for cancer diagnosis and therapy. Patients were classified into high- and low-risk groups based on the risk score, and the KM curve showed that the prognosis of patients in the low-risk group was much better than that of patients in the high-risk group. ROC analyses were performed to test the prediction efficiency of the risk score. Nomograms are widely used as prediction tools in oncology, particularly for survival prediction (Iasonos et al., 2008; Balachandran et al., 2015). A nomogram model was established according to the risk score and other clinical characteristics to accurately predict the survival time of patients, and the calibration plots showed that the actual survival rates were close to the nomogram-predicted survival rates. This indicated that the nomogram model had high accuracy in predicting patient survival.

The correlation between the risk score and immune cells was also analyzed; four types of immune cells were positively related to the risk score and the other 11 types of immune cells were negatively correlated with the risk score. The seven genes also showed significant associations with various types of immune cells. Dai et al. (2020) reported that an immune score based on immunogenomic analysis can indicate the efficacy of immunotherapy and chemotherapy. The high-risk group had higher stromal and lower immune scores, suggesting that the low-risk group might have a better response to antitumor therapy. CSCs are a subset of tumor cells associated with tumor metastasis, recurrence, and drug resistance. Similar to normal stem cells, CSCs exhibit self-renewal and differentiation abilities (Singh and Chellappan, 2014). The risk score was also related to the CSC index, indicating that the risk score may be related to colon cancer progression. The differences in immune checkpoint gene expression in the high-risk and low-risk groups were also analyzed, and the expression levels of checkpoints, including *CTLA4, LAG3, ID O 2, CD274*, and *PDCD1*, were found to be higher in the low-risk group. The correlation between risk groups and previously identified immune subtypes was analyzed; the results showed that the inflammatory samples were almost equally distributed between the two groups, but there was more wound healing and lymphocyte depletion and fewer IFN-gamma-dominant samples in the high-risk group than in the low-risk group. The IPSs of the two risk groups suggested that the low-risk group had a better response to *PD-1* and *CTLA-4* blockade therapy. IC50 values indicated that the low-risk group was more sensitive to immunotherapeutic and chemotherapeutic drugs, and the results confirmed our previous conclusion based on TME-related analyses. The findings of our study can be applied to guide clinical immunotherapy and chemotherapy in patients with colon cancer and help us to further understand the effects of PANoptosis on colon cancer. The expression levels of *LGR5*, *VSIG4*, *GZMB,* and *ITLN1* were further validated using qRT-PCR method, the results showed that *LGR5* was significantly upregulated in colon cancer while *VSIG4* was downregulated in colon cancer compared with normal tissues, indicating that *LGR5* and *VSIG4* may be potential biomarkers for diagnosis and therapy in colon cancer.

Nevertheless, our study has some limitations. Most analyses were based on data from public datasets, and all samples were obtained retrospectively, which may have caused an inherent case selection bias. In addition, limited molecular biology experiments were performed in the study, and further *in vitro* and *in vivo* experiments are needed to validate our findings. Finally, some valuable clinical features such as surgery, neoadjuvant chemotherapy, and tumor markers were not considered in our study. As such, clinical cases are needed to confirm our conclusions.

In summary, we constructed a PANoptosis-based molecular clustering and prognostic signature that plays a vital role in predicting survival, TMB, and guiding clinical therapy. The findings of this study may improve our understanding of PANoptosis in colon cancer and help develop more effective treatment strategies. However, this study has some limitations, and additional experiments and clinical cases are needed to validate our findings.

## Data Availability

The data presented in the study are deposited in the TCGA-COAD project and GEO database (accession number: GSE39582).
