# Supplementary material for: PANoptosis-based molecular clustering and prognostic signature predicts patient survival and immune landscape in colon cancer
Source: Front Genet. 2022 Sep 14;13:955355. doi: 10.3389/fgene.2022.955355 (PMC9515384; doi:10.3389/fgene.2022.955355)
Supplement: Supplementary file 2 [file Table3.DOC]

**Supplementary Table S3.** Primer sequences for PCR amplification.

| Genes | Forward Primer | Reverse Primer |
| --- | --- | --- |
| LGR5 | 5′-GCCTTCAATCCCTACATCTCCA-3′ | 5′-GAGAAGGGTTGCCTACAAATGC-3′ |
| VSIG4 | 5′-ATGGGGATCTTACTGGGCCT-3′ | 5′-GCCTGCTGGATATGGTCTCC-3′ |
| GZMB | 5'-GCAGACTTTTCCTTCAGGGGA-3' | 5'-GGTCGGCTCCTGTTCTTTGA -3' |
| ITLN1 | 5'-TCATAGCGACCACCAGAGGA-3' | 5'-CACGCATGTCATTCTCGTGC-3' |
| 36B4 | 5'-ATCCCTGACGCACCGCCGTGA-3' | 5'-TGCATCTGCTTGGAGCCCACGTT-3' |
