# Supplementary material for: PANoptosis-based molecular clustering and prognostic signature predicts patient survival and immune landscape in colon cancer
Source: Front Genet. 2022 Sep 14;13:955355. doi: 10.3389/fgene.2022.955355 (PMC9515384; doi:10.3389/fgene.2022.955355)
Supplement: Supplementary file 3 [file Table5.DOCX]

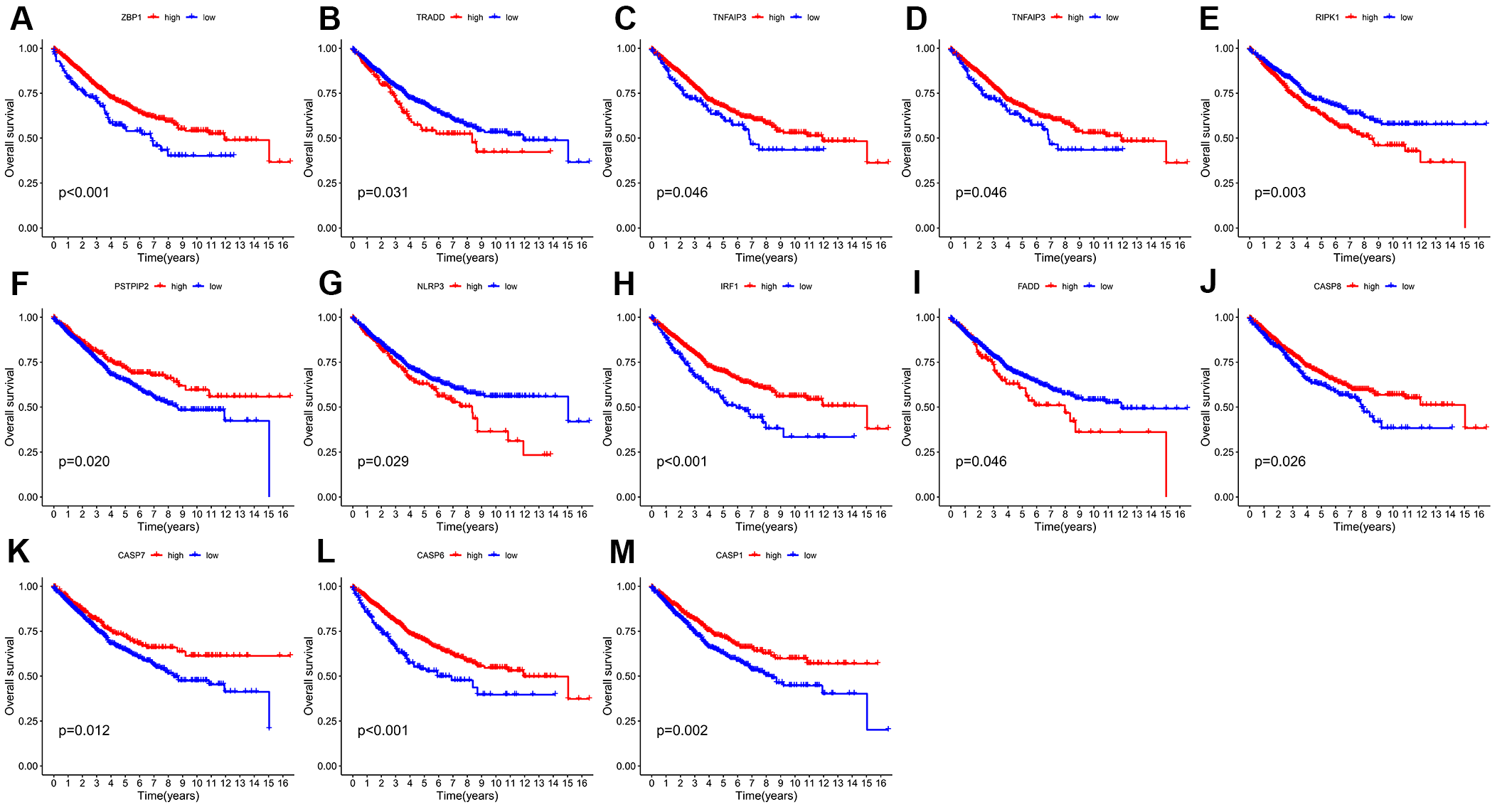


**Supplementary Figure S1:** Kaplan–Meier curves of the relationship between PRGs expression and the prognosis of colon cancer patients based on TCGA and GEO databases (*p* < 0.05).


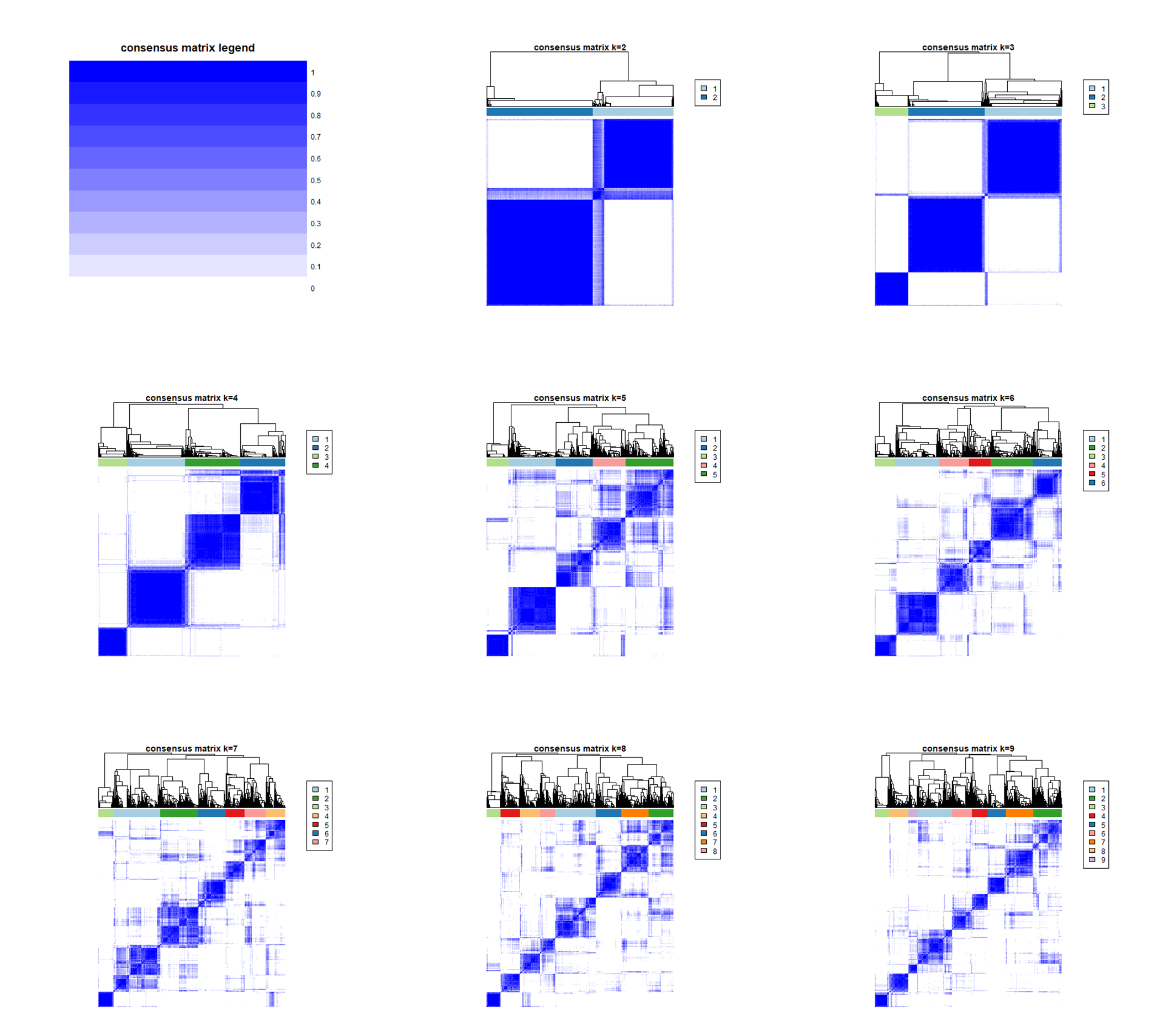


**Supplementary Figure S2:** Unsupervised clustering of PANoptosis-related genes and Consensus matrix heatmaps for k = 1-9.


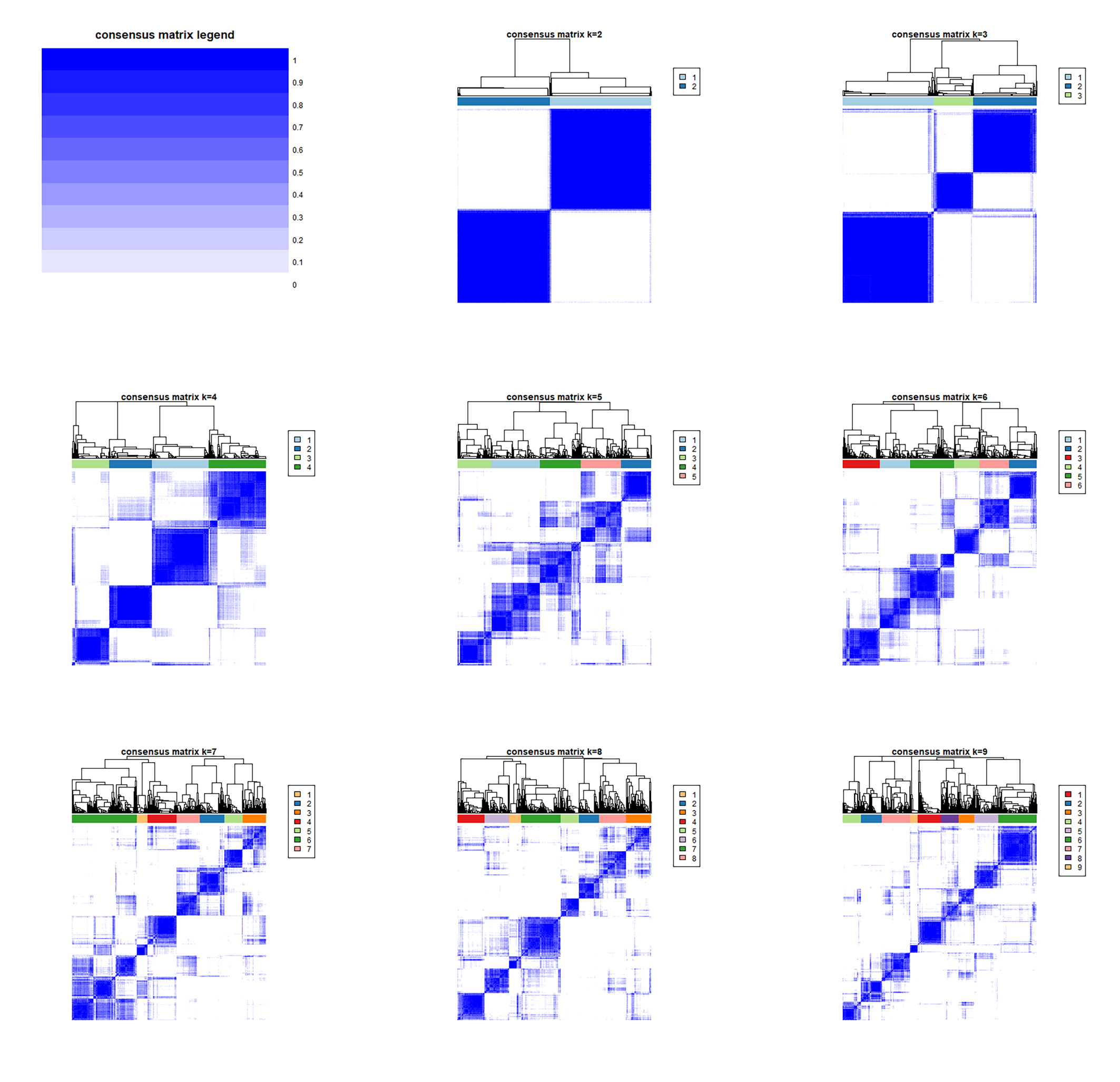


**Supplementary Figure S3:** Gene clustering and Consensus matrix heatmaps for k = 1-9.


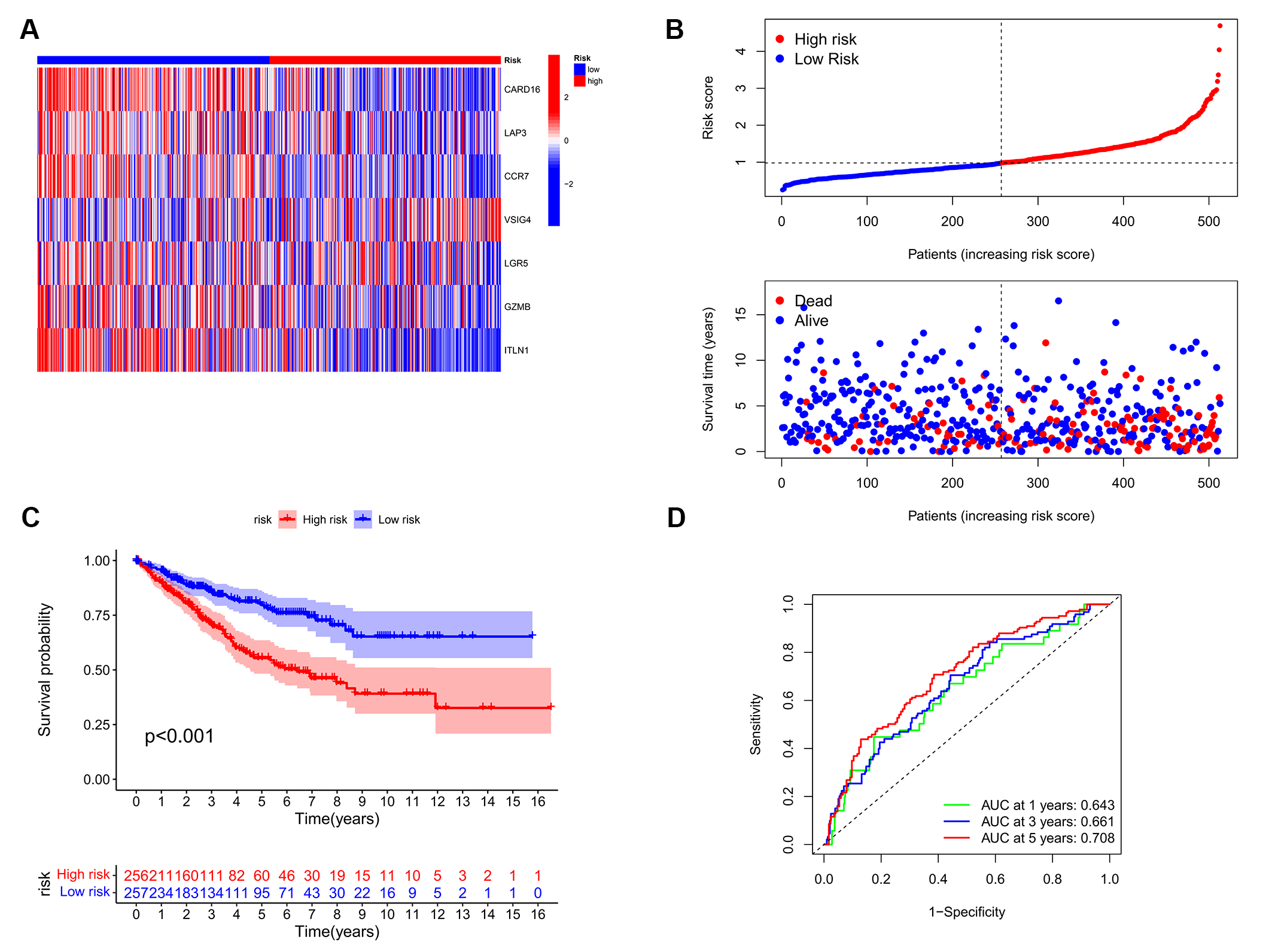


**Supplementary Figure S4:** Validation of the risk score in TCGA cohort. (A) Heatmap showed the expression of 7 genes in two risk groups. (B) Risk score and survival outcome of each case. (C) KM curve showed that patients in high-risk group had a worse prognosis (*p* < 0.001). (D) The AUC for 1-, 3- and 5-year survival were 0.643, 0.661, and 0.708, respectively.


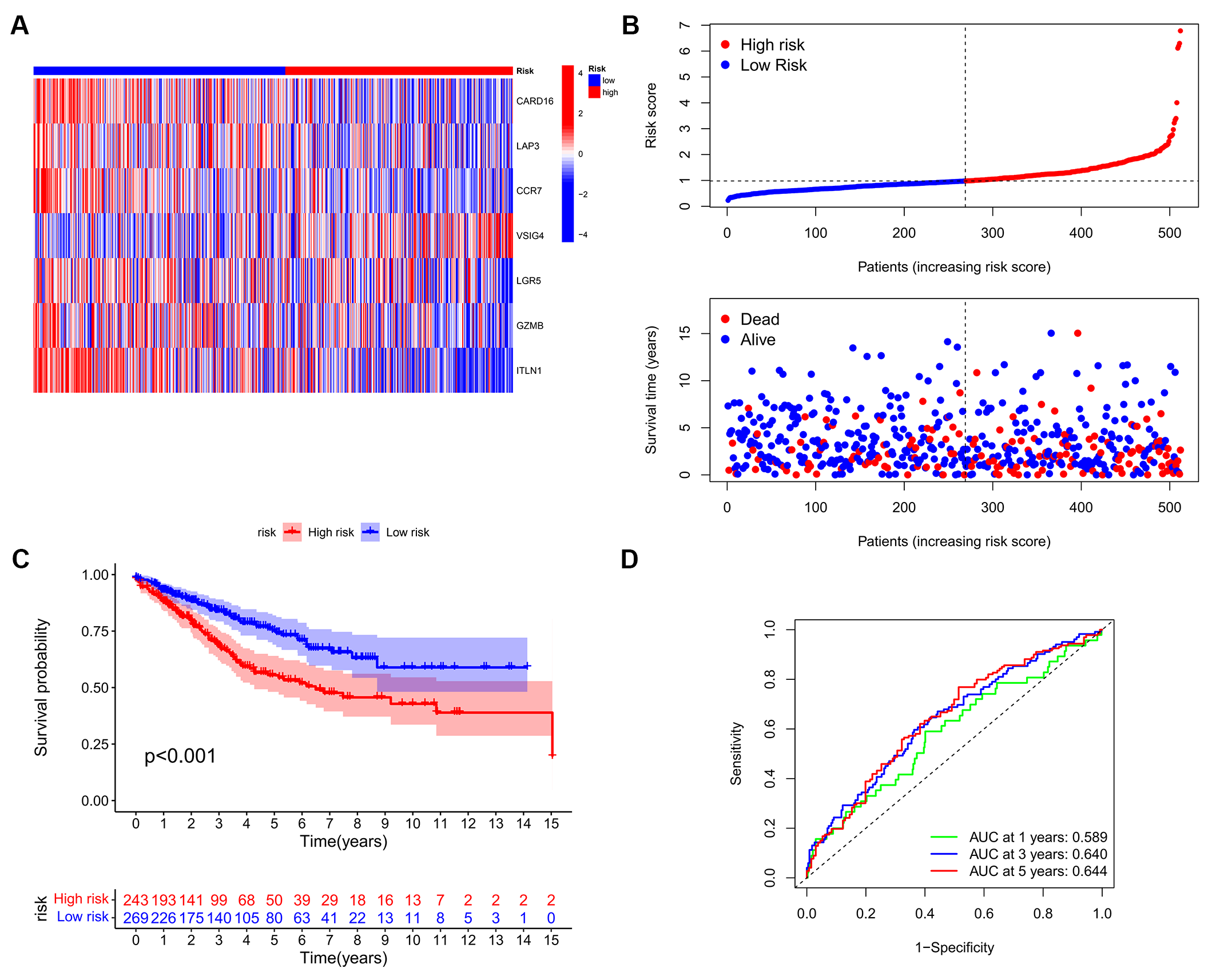


**Supplementary Figure S5:** Validation of the risk score in GEO cohort. (A) Heatmap showed the expression of 7 genes in two risk groups. (B) Risk score and survival outcome of each case. (C) KM curve showed that patients in high-risk group had a worse prognosis (*p* < 0.001). (D) The AUC for 1-, 3- and 5-year survival were 0.589, 0.640, and 0.644, respectively.


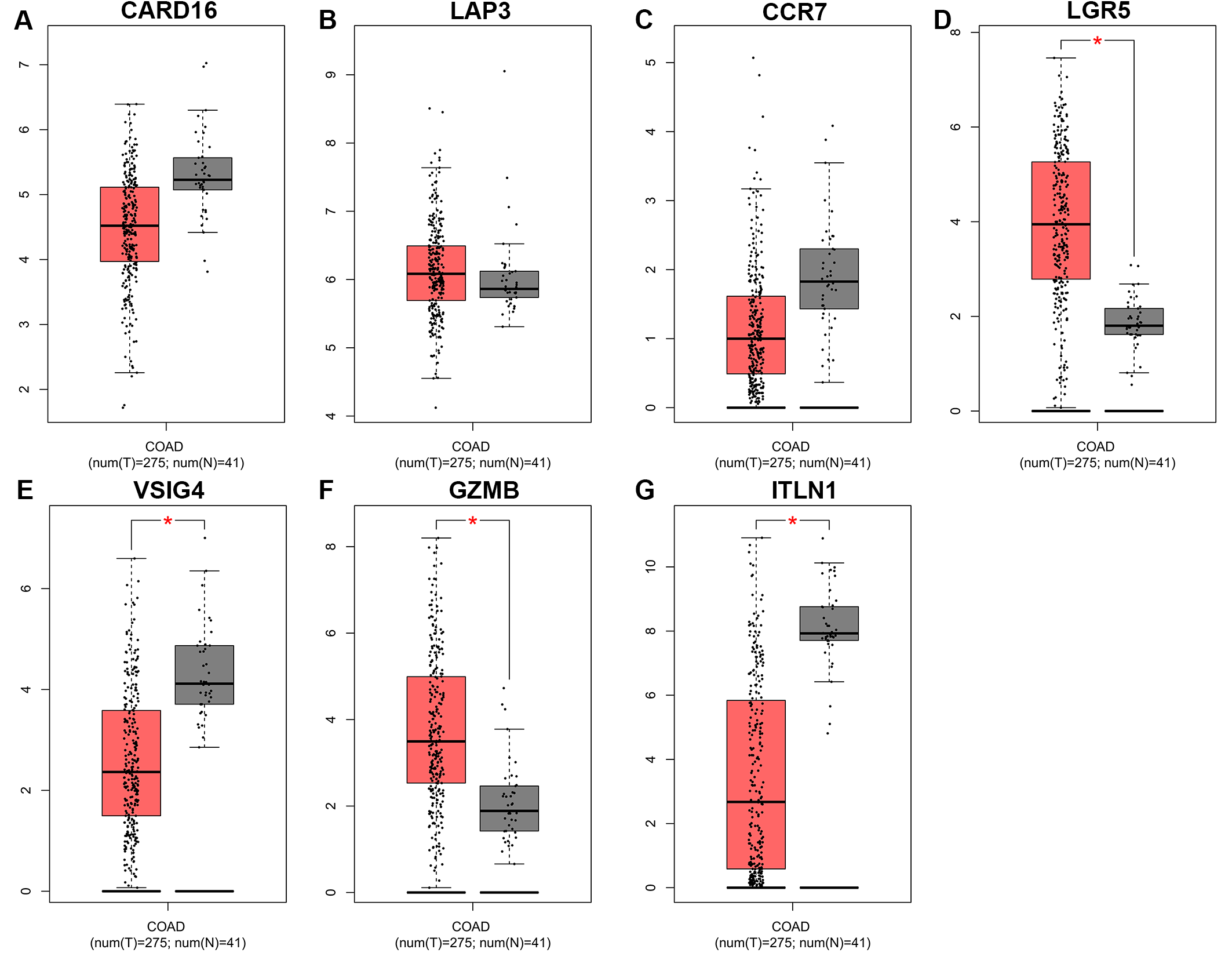


**Supplementary Figure S6:** Expression levels of CARD16 (A), LAP3 (B), CCR7 (C), LGR5 (D), VSIG4 (E), GZMB (F) and ITLN1 (G) in normal and colon cancer tissues (GEPIA database). Red represents tumor tissues while grey represents adjacent non-cancer tissues. **p* < 0.05.
